# What Is the Relative Value Unit of Being "On Call"?

**DOI:** 10.7759/cureus.2823

**Published:** 2018-06-18

**Authors:** Joseph J Stella, Kishoe Harjai

**Affiliations:** 1 Cardiothoracic Surgery, Geisinger Health System, Danville, USA; 2 Department of Cardiology, Geisinger Health System, Danville, USA

**Keywords:** relative value unit, on call

## Abstract

There is very little information on what is the relative value unit (RVU) for a physician being “on call.” This paper proposes a broad construct to devise a formula for the RVU of being on call.

## Editorial

Being “on call” is both a duty and a privilege of being a physician. The history of being “on call” and readily available for patients and their families at all times is part of the history and integrity of the noble profession. The images and stories of doctors making house calls in the middle of the night for numerous types of conditions and emergencies are well-sketched in our brains. As a matter of fact, many young individuals seeking a healthcare career are inspired by such stories. Without a doubt, our choice to enter medicine was guided by the aura of self-sacrificing doctors attending emergencies at odd hours, to alleviate suffering in patients and their families afflicted by clinical emergencies occurring after hours. For junior physicians, being on call was a great way to consult with senior physicians, build a practice, and earn a comfortable living. It was considered part of one’s duties to have practice privileges at the hospital.

In recent decades, the nature of the "noble profession" has evolved into the business of medicine. Hospitals, large multispecialty clinics, as well as insurers, have developed bottom-line approaches to minimize costs and maximize profits. Operating margins often dominate decisions by these entities. Yet, individual physicians often continue to provide free on-call services, largely guided by their altruistic beliefs stemming from a sense of belonging to a noble profession, where the doctor is expected to be selfless. For some specialties like primary care, the burden of being on call has been alleviated by the advent of hospitalist medicine. Yet, in many subspecialties, being on call remains an integral part of daily practice. Within this dichotomy, some physicians in specialties like surgery, neurosurgery, cardiology, and cardiothoracic surgery, expect to be paid by the hospitals for being on call or paid for call more than a stipulated number of nights. Hospitals have adjusted over time by employing full-time hospitalists and surgeons to provide emergency and after-hour services. As more physicians become employed and salaried and work effort and bonuses are based largely on RVUs (relative value units), there seems to be little mention of the concrete financial value of being on call. It is indisputable that being on call entails significant physical and emotional stress and takes a toll on the family life of doctors. It is our assertion that the "stress factor" associated with being on call is frequently overlooked from the perspective of physician reimbursement.

This paper proposes a broad construct to devise a formula for the RVU value of being on call. We performed a literature search on the RVU of being on call. Only two articles were found. One article reviews the components of the RVU and how radiology payment is calculated. It highlights trends in RVUs and the resultant payment for diagnostic and therapeutic imaging and examinations, and it discusses current issues involving RVUs and current procedural terminology codes [1]. The other article dealt with family physicians in a university practice using call RVUs to make being on call more equitable [2]. They devised a call RVU worksheet based on numerous factors and procedures to have a fair accounting of the amount and types of calls they were performing among the group of physicians. These articles do not correlate with the amount of time spent being available for specialties like cardiothoracic surgery, neurosurgery, interventional cardiology, or trauma/general surgery. Locum tenens agencies pay their doctors fair market value for participating in clinical care during routine hours and a different rate for being on call.

We propose the use of these fair market values to derive an RVU value for being on call. For example, in cardiac surgery, the going rate for locums is anywhere from $1800-$3000 per 24-hour-day coverage. A typical coronary artery bypass grafting (CABG) surgeon fee and RVU from the Medicare physician fee schedule on the Centers for Medicare and Medicaid Services (CMS) website is around $2000 and 40 RVUs. Thus, extrapolating this information, a 24-hour call should be valued at 40 RVUs and a 12-hour night call valued at 20 RVUs. More and more institutions are paying physicians for being on call or paying for being on call more than a stipulated number of nights per month. Institutions that need on-call coverage pay locum tenens agencies to find physicians to provide coverage. For salaried physicians, being on call is part of the compensation package. There is a dearth of information on how one could assign financial value to being on call. Hence, RVUs have been associated with patient care or procedures but are not systematically allocated for being on call. Our proposed construct is based on fair market value principles, utilizes readily available information, and represents an objective reference for dialogue between clinicians, physician leaders, and administrators.

There is scant information in the literature on the RVU value of being on call. Yet, the market value of serving as a locum tenens physician provides a reasonable construct to estimate the RVU value of being on call in one’s permanent job. We believe that being on call carries significant physical and emotional stressors, which are sometimes overlooked in the calculation of physician salaries. We propose a simple calculation to guide clinicians and organizational leaders in assigning a fair market value to on-call duties.
